# rTMS Improves Cognition in Patients With Self‐Limited Epilepsy With Centrotemporal Spikes With Electrical Status Epilepticus in Sleep via Increase of the Sleep Spindle

**DOI:** 10.1111/cns.70501

**Published:** 2025-07-07

**Authors:** Yixian Han, Jing Gao, Yongkang Zhou, Jiahui Deng, Huajun Yang, Xiongfei Wang, Jing Wang, Yujiao Yang, Jie Ren, Lingling Chen, Minghui Wang, Qinqin Deng, Haidan Wang, Mengyang Wang, Tianfu Li

**Affiliations:** ^1^ Department of Neurosurgery Sanbo Brain Hospital, Capital Medical University Beijing China; ^2^ Department of Neurology Sanbo Brain Hospital, Capital Medical University Beijing China; ^3^ Beijing Key Laboratory of Epilepsy Sanbo Brain Hospital, Capital Medical University Beijing China; ^4^ Beijing Institute for Brain Disorders Capital Medical University Beijing China; ^5^ Laboratory for Clinical Medicine Capital Medical University Beijing China

**Keywords:** cognition, electrical status epilepticus in sleep, repetitive transcranial magnetic stimulation, self‐limited epilepsy with centrotemporal spikes, sleep spindle

## Abstract

**Background:**

Patients with self‐limited epilepsy with centrotemporal spikes (SeLECTS) and electrical status epilepticus in sleep (ESES) lead to cognitive impairment. However, therapeutic options are limited.

**Objectives:**

The **s**leep spindle is a biomarker of cognitive dysfunction in patients with SeLECTS. This study aimed to explore whether the sleep spindle is linked to the outcome of repetitive transcranial magnetic stimulation (rTMS) in patients with SeLECTS with ESES.

**Methods:**

Nine patients with SeLECTS with ESES underwent low‐frequency rTMS (≤ 1 Hz) or continuous theta burst stimulation (cTBS) for 10 days. To assess the clinical efficacy and alteration in the sleep spindle, EEG recordings were performed both before and after rTMS. A machine learning algorithm YASA was used to calculate the coupling of the sleep spindle and slow waves.

**Results:**

75% of patients remained seizure‐free for 6 months after rTMS. The spike–wave index (SWI) decreased significantly after rTMS compared with the baseline. The sleep spindle significantly increased in all patients at 3 months and 6 months after rTMS (*p* = 0.002). Both IQ and MQ improved significantly at 6 months after rTMS. Improvement of IQ and increase of the sleep spindle was significantly positively correlated (*p* = 0.035). The mean probability of the sleep spindle coupling in the slow wave “up” state increased from 28% to 55%.

**Conclusion(s):**

Increase of the sleep spindle and the mean probability of the sleep spindle coupling in the slow wave “up” state might be a potential mechanism for cognition improvement in patients with SeLECTS with ESES.

## Graphical abstract

rTMS inhibits clinical seizure and decreases SWI. rTMS improves cognitive function. rTMS increases in the sleep spindle and the sleep spindle coupling in the slow wave up‐state. rTMS improves cognitive function via increases of the sleep spindle.

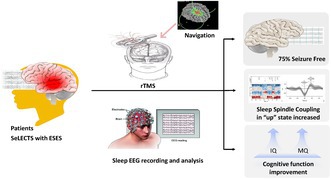

## 1 Introduction

Self‐limiting epilepsy with centrotemporal spikes (SeLECTS), previously referred to as “benign epilepsy of childhood with centrotemporal spikes” (BECT), and also known as “benign Rolandic epilepsy of childhood,” is the most common focal syndrome in childhood epilepsy [1]. The age of onset is typically between 3 and 13 years, with a peak incidence around 5–8 years. Spontaneous remission usually occurs about 2–4 years after onset [2]. This condition affects 15%–25% of children, with a male predominance [3]. In May 2022, the International League Against Epilepsy formally proposed removing the term “benign” from the names of epilepsy syndromes, favoring the term “self‐limited” instead, and BECT was renamed SeLECTS in the latest classification because the typical evolution of this type of epilepsy is age‐related onset and remission [4, 5]. Most patients have normal psychomotor development, and neuroimaging typically shows no positive findings. Seizures often present with unilateral symptoms such as mouth twitching, salivation, dysarthria with reserved awareness, which can be accompanied by convulsions in the ipsilateral upper limb. The seizures can develop into bilateral tonic–clonic seizure with impaired awareness [4]. A small percentage of these patients have frequent seizures, or atypical seizures‐negative myoclonus, atypical absence, opercular syndrome or even epileptic encephalopathy. These patients show electrical status epilepticus during sleep (ESES), in which alterations in the salience network and central executive network can be observed [6].

ESES patterns usually consist of continuous, symmetrical spike patterns with variable discharge frequencies (1.5–3 Hz usually). The spike–wave index (SWI), the percentage of the spike and slow wave duration to the total non‐REM (NREM) sleep duration, is commonly used to measure the severity of ESES [7]. SWI ≥ 50% could trigger the occurrence of ESES [8]. In the current study, we defined SWI ≥ 50% as ESES. A significant decrease in cognitive function was indicated in the SWI ≥ 50% group compared with the SWI < 50% and healthy control group [9]. Nearly continuous epileptiform activity during slow wave sleep often results in significant declines in cognitive or behavioral function, involving language, communication, temporospatial orientation, attention, and social interaction [10]. Epilepsy is gradually controlled after puberty, but varying degrees of cognitive impairment may persist, indicating that the clinical course is not entirely “benign”.

SeLECTS with ESES tends to develop into refractory epilepsy, and a longer period of oral glucocorticoid maintenance is needed in order to control the seizures. Once the medicine is discontinued, it will lead to recurrence of epilepsy. rTMS is a widely used non‐invasive neuromodulatory technique [11]. Low‐frequency rTMS (≤ 1 Hz) inhibits cortical excitability, prolongs the cortical silent period, and reduces the motor evoked potential amplitude [12]. Continuous theta burst stimulation (cTBS), which embeds a 50 Hz pulse within a 5 Hz pulse, also serves as an inhibitory therapeutic parameter. rTMS is indicated as a promising approach to reducing clinical seizures for BECTS with frequent seizures [13]. rTMS was indicated to modulate the nocturnal sleep spindle activity [14]. The sleep spindle, as a physiological indicator of intelligence, is highly correlated with intelligence tests and the function of the sleep spindle is related to intellectual ability and memory consolidation [15]. We hypothesized that rTMS may offer efficacy for treating SeLECTS with ESES and improve the patient's intelligence and memory via modulation of the sleep spindle.

## 2 Materials and Methods

### 2.1 Patient Selection

The study was approved by the Ethics Committee of the Sanbo Brain Hospital, Capital Medical University. Nine children were diagnosed with SeLECTS with ESES at the Comprehensive Epilepsy Center, Sanbo Brain Hospital from August 2015 to July 2022. The median age was 7 (5.0, 8.5) years, including five males (56%) and four females (44%). The inclusion criteria were as follows: (i) age of onset between 3 and 15 years; (ii) clear diagnosis of self‐limited epilepsy with centrotemporal spikes; (iii) SWI ≥ 50%; (iv) refractory epilepsy or epileptic encephalopathy. Patients with a history of prematurity (< 35 weeks), abnormal magnetic resonance imaging (MRI) of the brain, other epilepsy syndromes, neurosurgery, or severe brain injury were excluded. All the patients received follow‐up for at least 6 months after TMS.

### 2.2 Repetitive Transcranial Magnetic Stimulation

**FIGURE 1:**
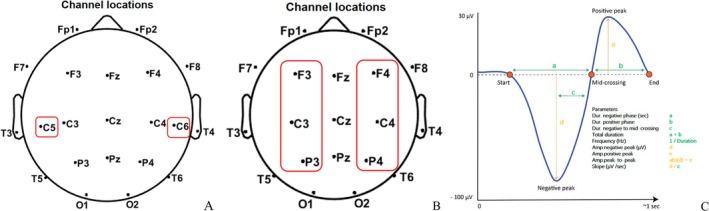
rTMS treatment localization and schematic diagram for slow wave‐sleep spindle coupling. (A) The therapeutic sites in seven patients were localized to C5 or C6 (red box) according to EEG discharges and semiology. (B) The location of the sleep spindle recording was shown in the bilateral frontal, central, and parietal regions (F3/F4, C3/C4, P3/P4) (red boxes). (C) Schematic diagram (Courtesey from R.Vallat) for slow wave‐sleep spindle coupling demonstrates as follows: Duration of the negative phase (a), duration of the positive phase (b), duration from the negative peak to the point past zero (c), duration of one epoch for slow wave identification and frequency (a + b), the amplitudes of the negative peak (d) and the positive peak amplitude, the peak‐to‐peak value (abs(d) + e). The slope is defined as the value from the negative peak to the point past zero.

**FIGURE 2:**
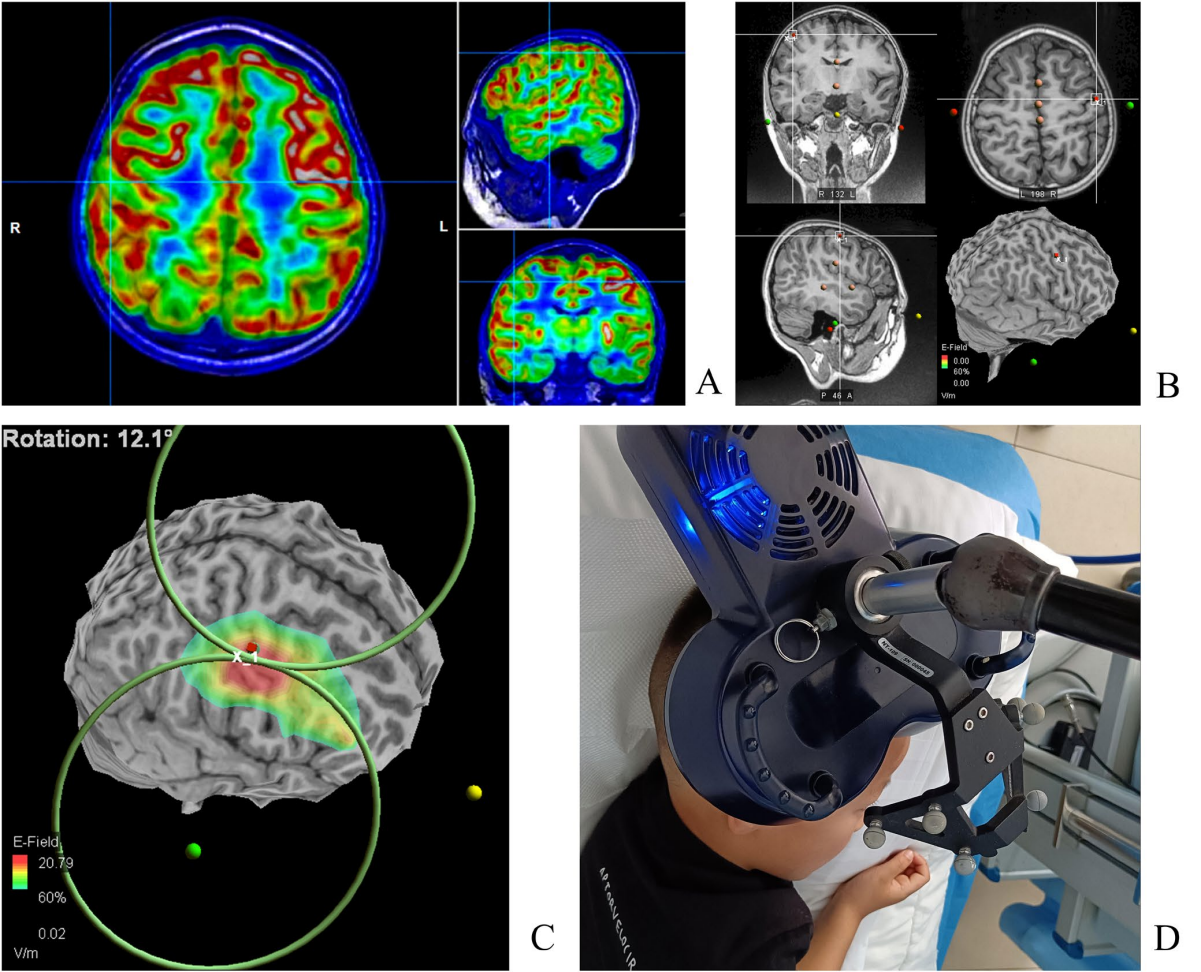
Location of treatment by rTMS‐specific navigation. (A) Fusion of T1‐weighted MRI and the PET map revealed a hypometabolic region in the right central area (crosshairs intersecting). (B) The same set of T1 MRI images was input into the special TMS navigator for reconstruction of three‐dimensional cortical reconstruction. The hypometabolic region was localized as the therapeutic site (3D rendering of the right hemisphere with target site marked by red dot). (C, D) The coil was guided by the navigation system and used to stimulate the hypometabolic site at a low‐frequency stimulation (0.5 Hz, 40% output intensity). Treatment protocol consisted of 10 daily sessions with 1000–1500 pulses administered per day.

In the early stages (2015–2019), the localization of the treatment was determined based on the clinical seizure presentation and EEG discharge distribution. The EEG international 10–10 system was used to localize C5/C6 (central facial movement representative area) (Figure 1A). After 2020, the localization was determined with TMS‐specific navigation software for three‐dimensional reconstruction of MRI images (Figure 2). This involved comparing image data from PET‐CT and MRI fusion with navigation data to localize hypometabolic or hypermetabolic zones adjacent to the facial representation of the central region. Low‐frequency stimulation (0.3, 0.5, and 1 Hz) by Rapid 2 (Magstim) was utilized based on the frequency of seizures and the patient's cooperation at the beginning of treatment. Eight patients received 10 workdays of stimulation with 1000–1500 pulses administered per day. The cTBS treatment modality by YD‐MT600 (Neurosoft) was used in one patient. Stimulation intensity for rTMS treatment was set at 40%–60% according to our previous clinical experience and the patient's tolerance. The ASMs remained constant in all patients during the rTMS treatment without the other therapeutic interventions (Table 1).

**TABLE 1:** Lateralization, localization and parameter selection information of rTMS treatment for all patients with SeLECTS with ESES.

Pt/No.	rTMS stimulus parameters				Stimulation site choose		
	Site	Intensi	Frequency	Number of stimuli	Lateralization by semiology	EEG discharges	Set side
1	C6	40%	1 Hz	1500	Left	Bilateral, prominent on the right side	Right
2	C5	40%	0.3 Hz	1500	Right	Bilateral	Left
3	C6	40%	0.33 Hz	1000	Weak in lateralization	Right side	Right
4	C6	40%	0.33 Hz	1500	Left	Bilateral, prominent on the left side	Right
5	C6	50%	0.5 Hz	1500	Left	Bilateral, prominent on the right side	Right
6	C6	50%	1 Hz	1500	Left	Bilateral, prominent on the right side	Right
7	Left PET Hypermetabolism	50%	0.5 Hz	1000	Weak in lateralization	Bilateral	Bilateral
8	C6	60%	cTBS	600	Left	Right side	Right
9	Right PET Hypometabolism	50%	0.5 Hz	1000	Left	Bilateral, prominent on the right side	Right

### 2.3 EEG Recordings

**FIGURE 3:**
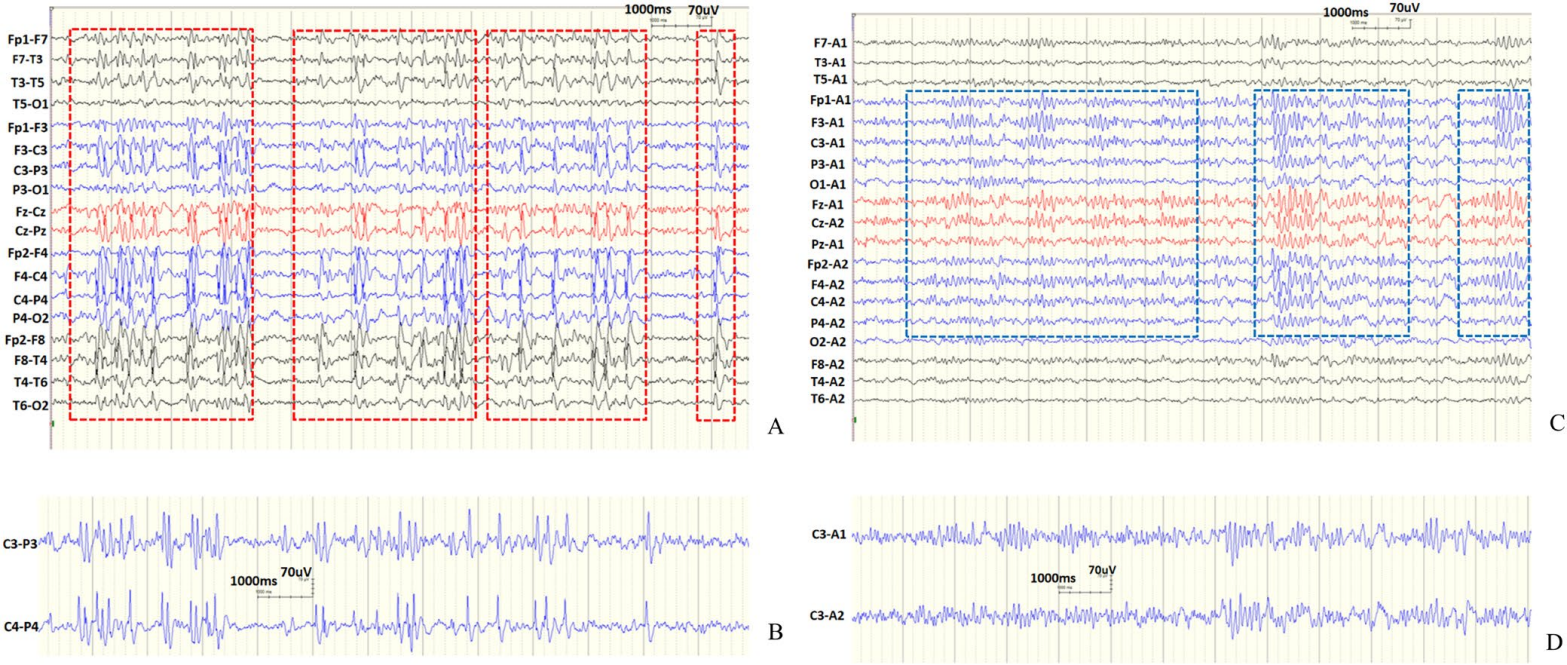
rTMS reduces epileptiform discharges and restores sleep spindle activity in Case 1. (A) Nearly continuous discharges were observed in both hemispheres during sleep before rTMS treatment (highlighted in the red box), with no sleep spindle wave forms present. (B) Spikes in C3 and C4 were demonstrated. (C) After rTMS treatment, the patient became seizure‐free, with epileptiform discharges completely eliminated and the background activity of EEG during sleep fully recovered after 6 months. The sleep spindle (highlighted in the blue frame) was visible in frontal, parietal, and central channels, as well as in the ear electrode reference montage. (D) The Sleep spindle in C3 and C4 was demonstrated.

EEG recordings were conducted using the standard international 10–20 system, employing a total of 19 scalp electrodes. The Nicolet recording system was used for EEG recordings during both sleep and wakefulness. SWI was reviewed by an independent electrophysiologist who guaranteed the confidentiality of clinical data. The sleep spindle was analyzed during the first 10 min of stage II sleep in the first sleep cycle. The sleep spindle, with a frequency of 10–15 Hz, was counted in the frontal, central, and parietal lobe using A1/A2 reference leads. Instances of synchronization between both hemispheres were counted once, while asynchronous occurrences with a time difference exceeding 1 s were counted twice, with the longest time course recorded. Manual counting was adopted due to significant discrepancies between initial trial software calculations and manual counts. Two senior electrophysiology experts independently conducted the manual counting, with discrepancies less than 3 being accepted without re‐counting. In cases of inconsistent results with discrepancies exceeding 3, additional counts and discussion were undertaken, and results were confirmed after reaching consensus (Figures 1B and 3).

### 2.4 Slow Wave‐Sleep Spindle Coupling

A machine learning algorithm utilizing YASA was employed. The slow wave screening frequency was set to 0.5–4 Hz, with a down duration range of 0.18–1.5 s and an up duration range of 0.06–1 s. The sleep spindle screening frequency was set at 10–15 Hz, lasting 0.5–4 s. The coupling identification time window was set as 1 s before and after the crest of the down portion of the slow wave. Slow waves and the sleep spindle were monitored during sleep phase II, with the coupled sleep spindles being sought by the YASA package among the identified slow waves. The coupled entries were conditioned, and the final number of results was obtained [16] (Figure 1C).

### 2.5 Neuropsychological Evaluation

All children underwent assessment using standard evaluation procedures. Intelligence was assessed before and after treatment for all patients, while memory was evaluated in children aged over 7 years. The Wechsler Intelligence Scale for Children, fourth edition (WISC‐IV), was utilized to measure the intelligence level of the children, while the Wechsler Memory Scale was employed to evaluate the memory level.

### 2.6 Follow‐Up Program

Seizure frequency, SWI, sleep spindles, and WISC‐IV were assessed in nine patients, and Wechsler Memory Scale was assessed in five patients. Seizure frequency, SWI, and the sleep spindle were assessed at 3 months and 6 months after treatment. Wechsler Memory Scale, WISC‐IV, and a slow wave and the sleep spindle coupling were assessed at 6 months after treatment.

### 2.7 Statistical Analysis

All data were tested for normality. Normally distributed data were expressed as X ± S, while non‐normally distributed data were expressed as M (P25, P75), and counts were expressed as rate (%). Because of the small sample size, normality was determined by Shapiro–Wilk, and the values of sleep spindle and MQ in all groups conformed to normal distribution, and a Paired‐Samples *t* test was used. Values for seizure frequency, SWI, and IQ that did not exhibit a normal distribution were analyzed via Wilcoxon signed‐rank test, a nonparametric test. Correlation analysis was performed using the Spearman correlation coefficient. Comparative data for seizure frequency, SWI, and sleep spindle in pre‐treatment, 3‐month post‐treatment, and 6 months post‐treatment were subjected to Benjamini‐Hochberg (FDR) validation. All test *p*‐values less than 0.05 were considered statistically significant.

## 3 Results

### 3.1 Clinical Information

Nine patients (four females and five males) with SeLECTS with ESES were enrolled. Eight of the patients had refractory epilepsy, and one had epileptic encephalopathy. Median age was 7 (5.0, 8.5) years. MRI results were normal for all patients. Seven patients presented salivation during non‐seizure episodes, including one case with opercular syndrome. Before rTMS, the median number of seizures per month was 5 (1.13, 26.25), median SWI was 81% (72.5%, 89.5%) and mean number of the sleep spindle was 54.56 ± 43.97. Detailed information is provided in Table 2.

**TABLE 2:** Demographic characteristics and medical history of all patients with SeLECTS with ESES.

Pt/No.	Gender	Age at rTMS	MRI	Frequency (Seizures per month)	SWI before rTMS(%)	Operculum syndrome	Constant salivation	Sleep spindles
1	Male	5	Normal	3	80	No	Yes	116
2	Male	9	Normal	0	50	No	No	97
3	Male	5	Normal	15	81	No	Yes	73
4	Female	8	Normal	6	86	No	Yes	8
5	Female	7	Normal	4	81	No	Yes	58
6	Male	5	Normal	300	90	Yes	Yes	8
7	Female	9	Normal	0.5	89	No	No	9
8	Female	7	Normal	0.1	90	No	Yes	22
9	Male	5	Normal	30	65	No	Yes	100

### 3.2 Seizure Frequency Before and After rTMS

**FIGURE 4:**
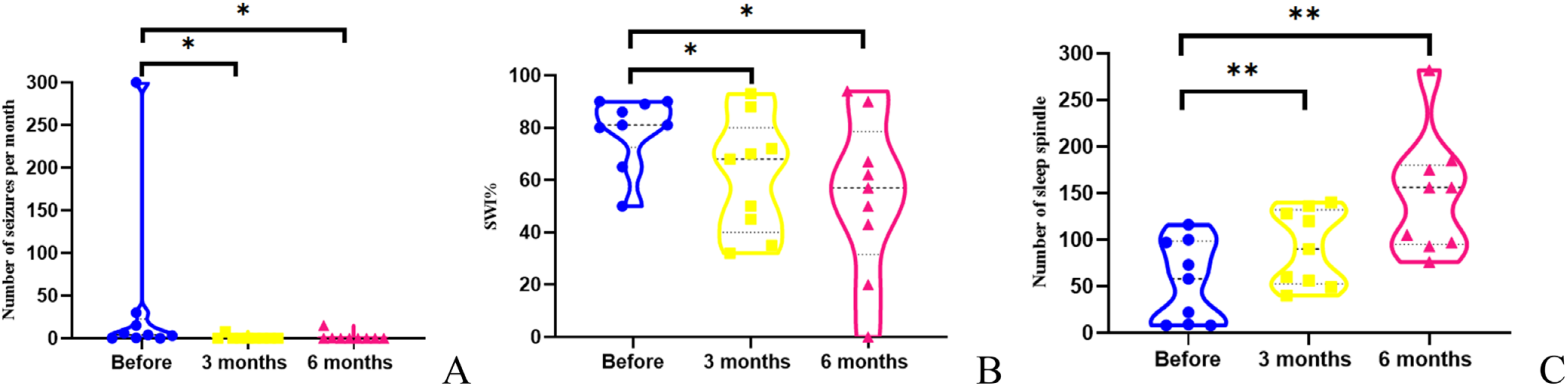
rTMS reduces seizure frequency and SWI while increasing sleep spindle density. (A) Six patients (75%) were seizure‐free, and the patient's seizure frequency markedly reduced at 3 and 6 months post‐treatment compared to pre‐treatment (*p* = 0.024, *p* = 0.018 respectively). (B) Compared to before rTMS, SWI decreased at 3 months and 6 months after rTMS (*p* = 0.045, *p* = 0.035 respectively). (C) The sleep spindle significantly increased in all patients at 3 months and 6 months after treatment (*p* = 0.002, *p* = 0.002 respectively) (All *p*‐values are FDR adjusted. *p < 0.05, **p < 0.01.).

All patients completed rTMS treatment. The overall number of seizures per month significantly decreased at 3 months and 6 months after rTMS (Table 3). Six out of the eight patients (75%) were seizure‐free. Notably, these six patients were also seizure‐free before treatment, and the data of patient 2 were not included in the seizure frequency analysis. One patient experienced a 60% reduction at 3 months and a 40% reduction at 6 months in seizure frequency. The other experienced a 47% reduction in seizure frequency at 3 months (*p* = 0.024) and returned to baseline at 6 months after rTMS (*p* = 0.018) (Figure 4A).

**TABLE 3:** Comparison of seizure frequency before and after 3 months and 6 months rTMS treatment.

Groups	Total number of seizures per month	M (P25, P75)
Before rTMS	358.6	5 (1.13,26.25)
3 months after rTMS	8.2	0 (0, 1)
6 months after rTMS	15.3	0 (0,0.23)

### 3.3 SWI Before and After rTMS

All the patients had SWI ≥ 50%. The median SWI after rTMS decreased from 81% (baseline) to 68% at 3 months and then to 57% at 6 months. The SWI at 3 months was significantly reduced by rTMS (*p* = 0.045). The SWI at 6 months significantly decreased in six patients, mildly increased in two patients, and remained unchanged in one patient after rTMS (*p* = 0.035) (Figure 4B).

### 3.4 Sleep Spindle Before and After rTMS

There was a significant increase in the sleep spindle of all patients at 3 months after rTMS (*p* = 0.002) and 6 months (*p* = 0.002); the mean number of the sleep spindle increased from 55 to 91 at 3 months and further increased to 147 at 6 months after rTMS (Figure 4C). The changes in the sleep spindle at 6 months after rTMS were observed in five aspects (Table 4): (i) All patients showed an increased number of sleep spindles; (ii) Longest single duration increased in seven patients (78%), decreased in one patient (11%) and was unchanged in one patient (11%), with the mean duration increasing from 1.87 s to 2.84 s; (iii) In the frequency range of 10–12.5 (Hz), the number of the sleep spindle increased in all patients; and the mean number of the sleep spindle increased from 33 to 79 before and after rTMS. In the frequency range of 12.6–15 (Hz), the number of the sleep spindle increased in eight patients (89%), and the mean number of the sleep spindle increased from 22 to 69; (iv) Seven patients (78%) showed an increase while two patients (22%) showed a decrease in maximum amplitude, and the mean maximum amplitude increased from 89 μV to 101 μV; (v) Seven patients (78%) showed improvement in amplitude modulation while two patients (22%) showed no alteration.

**TABLE 4:** Spindle characteristics before and after treatment 6 months in patients with SeLECTS with ESES (before/after).

	Duration (s)			Longest single duration (s)	Frequency (Hz)		Maximum amplitude (uV)	Amplitude modulation
	0.5–0.9	1–1.5	> 1.5		10–12.5	12.6–15		
1	103/102	10/61	3/22	2.1/3.6	64/58	52/127	203/168	Bad/fine
2	43/93	38/44	16/19	1.9/2.8	41/101	56/55	52/67	Poor/poor
3	55/57	13/32	9/16	2.1/2.8	55/76	18/29	77/74	Bad/good
4	5/105	2/47	1/23	2.7/2.5	8/108	0/67	84/89	Bad/fine
5	51/124	7/84	0/74	1.1/4.7	47/185	11/97	87/102	Bad/fine
6	7/74	1/14	0/5	1.4/1.9	1/3	7/90	62/86	Bad/poor
7	8/69	1/16	0/12	1.0/2.0	0/65	9/32	45/64	Bad/good
8	17/64	4/11	1/1	1.6/1.6	16/30	6/46	60/107	Bad/poor
9	29/27	40/33	31/96	2.9/3.7	62/82	38/74	134/149	Poor/poor
Mean	—	—	—	1.87/2.84	33/79	22/69	89/101	—

### 3.5 Neuropsychological Evaluation

**FIGURE 5:**
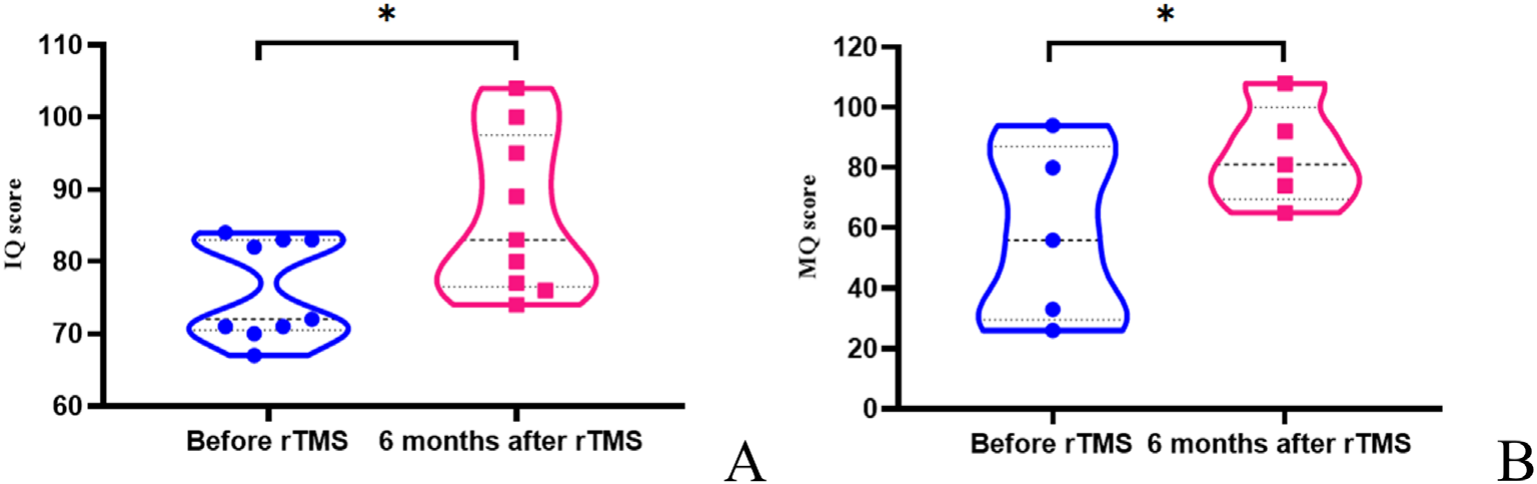
rTMS improves IQ and MQ in patients with SeLECTS‐ESES. (A) All patients underwent IQ assessment before and after treatment. The IQ of all patients was lower than the normal range. The IQ of all patients increased significantly at 6 months after rTMS (*p* = 0.012). (B) Five patients aged ≥ 7 years underwent a memory assessment. Before treatment, the MQ of the five patients was lower than the normal range. The MQ increased significantly at 6 months after rTMS (*p* = 0.016). *p < 0.05.

All patients underwent IQ assessment, and five patients more than 7 years old also underwent memory assessment before rTMS and at 6 months after rTMS. All IQ and MQ scores were below the normal range before treatment. After rTMS, the median IQ increased from 72 to 83. The IQ of nine patients increased (*p* = 0.012), and the MQ of the five patients increased after stimulation (*p* = 0.016) (Figure 5). Improvement in IQ and increase in the sleep spindle were significantly positively correlated (*p* = 0.035).

### 3.6 Slow Wave‐Sleep Spindle Coupling Before and After rTMS

**FIGURE 6:**
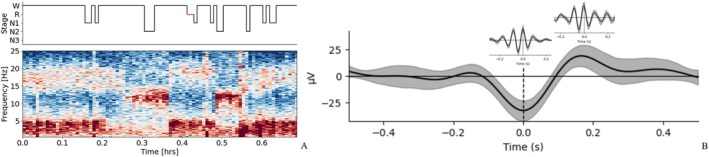
Slow wave‐sleep spindle coupling analyzed by YASA algorithm. According to the YASA algorithm, the schematic diagram illustrates the coupling waveform of the slow wave and sleep spindle. (A) W indicates the wake state and R indicates REM stage, illustrating the time‐frequency analysis, with the corresponding slow wave component visible in stage N2. (B) The two sub‐graphs depict the spindle waveforms coupled at different positions of 51 slow waves, respectively. The number of the sleep spindle coupled to the up and down states is 33 and 18 respectively (case 6).

The number of slow wave‐sleep spindle coupling before and after rTMS was recorded for all the patients. The probability of spindle coupling in the slow wave “up” state was analyzed. The mean probability of the sleep spindle coupling in the slow wave “up” state increased from 28% to 55% at 6 months after rTMS (Figure 6).

### 3.7 Improvement in Other Clinical Symptoms

Seven patients presented salivation during non‐seizure episodes before rTMS. Salivation symptoms during non‐epileptic episodes improved in five patients (disappeared in 4 and improved in 1), while the other two patients showed no alterations after rTMS. The symptoms of opercular syndrome in one patient showed significant alleviation. The symptoms of significant memory loss, irritability, hyperactivity, and inattention in another patient improved. No adverse effects were reported during or after rTMS.

## 4 Discussion

### 4.1 rTMS Inhibits Seizures, Decreases SWI and Improves Cognition

SeLECTS is a well‐defined electroclinical syndrome characterized by focal seizures and typical EEG abnormalities [17]. 4.6%–5.8% of such patients will experience atypical evolution during the course of the disease, including ESES, Landau–Kleffner syndrome (LKS), motor dysfunction, refractory epilepsy, etc. [18]. Rolandic double/multiple spikes and slow‐wave abnormalities in the interictal period may indicate ESES in SeLECTS [19]. Both atypical and typical ESES exhibit cognitive and executive function impairment [20]. Most of those with SeLECTS and ESES are refractory epilepsy patients with limited options to reduce seizures and improve cognitive‐behavioral function. Currently, the optimal treatment regimen has not yet been established.

TMS utilizes electromagnetic coils to excite or inhibit neurons, and low‐frequency repetitive pulses produce an inhibitory effect that reduces cortical excitability [21]. Extensive studies have demonstrated that rTMS yields significant clinical improvements in patients with various neurological and psychiatric disorders [22]. rTMS in both the ictal and interictal states, over the seizure focus in patients with focal epilepsy, could abort seizures and reduce seizure frequency or severity [23]. Low‐frequency rTMS is effective and safe in the treatment of drug‐resistant epilepsy [24]. Our previous study indicated that 62.5% of patients with SeLECTS with ESES got seizure‐free and SWI significantly decreased within 3 months after stimulation [10]. In the present study, 75% of patients with SeLECTS with ESES achieved seizure‐free. In addition, a significant reduction of SWI was found from 81% to 68% and 57% at 3 months and 6 months after rTMS respectively. It is important to note that rTMS alleviated symptoms of persistent salivation, opercular syndrome, and epileptic encephalopathy in patients with SeLECTS with ESES. It is well known that differences in the parameters of rTMS lead to differences in efficacy. High‐frequency (> 5 Hz) rTMS is thought to induce excitatory effects on the cortex, while low‐frequency (< 1 Hz) stimulation is considered to induce inhibitory effects. Whereas cTBS generally reduces cortical excitability [25], cTBS and low‐frequency rTMS are thought to cause MEP amplitude reduction [26]. Low‐frequency stimulation of rTMS was used in 8 of the 9 patients in this study. One patient with poor cognition could not cooperate with the comparatively longer‐term treatment, so the patient accepted cTBS therapy. Both therapies have similar mechanisms of action and efficacy and could not impact the reliability of the results.

In the present study, all patients with SeLECTS with ESES had cognitive impairment indicated by a decrease in IQ. rTMS demonstrated various degrees of cognition improvement.

### 4.2 rTMS Increases Sleep Spindles and the Sleep Spindle Coupling in the Slow Wave Up‐State

The sleep spindle is generated in the reticular nucleus of the thalamus, comprising a diffuse network of GABAergic neurons that encase other thalamic nuclei [27]. The sleep spindle appears as 10–15 Hz sinusoidal cycles and serves as a marker of the N2 stage of non‐REM sleep on EEG. The sleep spindle reflects the strength and malleability of thalamocortical circuits, and its density correlates with markers of intelligence, as well as various cognitive deficits and dementia‐related disorders [28]. Abnormal electrophysiology associated with sleep suggests disturbances in thalamocortical circuits, wherein dysfunction may hinder the generation of the sleep spindle, which is a brain rhythm crucial for memory processes [29]. Memory restoration occurs during the sleep spindle events and is subsequently reprocessed during spindle refractory periods [30]. Interventions that increase spindle activity have been shown to enhance sleep‐dependent memory consolidation [31, 32, 33]. Meta‐analyses revealed a positive relationship between the sleep spindle and overall cognition. A decrease in the sleep spindle is a predictive biomarker for cognitive impairment [34]. Our data showed a significant increase in the sleep spindle of all patients after rTMS. The mean number of sleep spindles increased from 55 to 91 and 147 at 3 months and 6 months, respectively, after rTMS. rTMS modulates the sleep spindle in duration, frequency, amplitude, and amplitude modulation in the majority of cases. Of most importance, improvement in IQ and increase in the sleep spindle were significantly positively correlated in the present study. By analyzing the alteration of the sleep spindle and associated neuropsychological performance after treatment, we postulated that an increase in the sleep spindle might be a potential mechanism for cognitive improvement in patients with SeLECTS with ESES.

Furthermore, we explored the role of rTMS on the coupling of the sleep spindle and slow waves. Slow wave‐sleep spindle activation in the hippocampus is indicated to consolidate memories during sleep [35]. During brain maturation from childhood to adolescence, the precision of slow oscillation and the sleep spindle coupling increases, contributing notably to enhanced consolidation in declarative memory tasks, recall, and sleep dependence. This phenomenon may serve as an indicator of the development of the hippocampal–cortical memory system [36]. Additionally, functional coupling between hippocampal ripples and thalamocortical sleep spikes and slow waves during NREM sleep facilitates fine‐tuned communication between the hippocampus and neocortex, thereby benefiting memory consolidation [37]. The timing of precise coordination between the NREM slow oscillation “up” state and the sleep spindle predicts the success of hippocampus‐dependent memory consolidation [38]. The slow‐oscillation “up” state is necessary for the replay and possible consolidation of previously learned tasks or experiences [39]. Our results revealed that the mean probability of the sleep spindle coupling in the slow wave rising “up” state increased from 28% to 55% at 6 months after rTMS. The sleep spindle coupling in the slow wave “up” state might be a potential mechanism for cognition improvement in patients with SeLECTS with ESES.

## 5 Conclusion

In conclusion, as a non‐invasive therapy, rTMS reduced seizure frequency and SWI and improved cognitive functions in patients with SeLECTS with ESES. In addition, rTMS increases the number of sleep spindles and the probability of the sleep spindle coupling in the slow wave “up” state. In the present study, an increase in the sleep spindle as well as the sleep spindle coupling in the slow wave up‐state may contribute to cognitive improvement.

## 6 Limitations and Future Directions

We acknowledge limitations to the interpretation of these results. Because we analyzed a relatively small cohort of patients, we cannot exclude the influence of clinical variables. Moreover, without a pseudo‐stimulation group, it is difficult to exclude the possibility of time or placebo effects influencing the results. Consequently, future research with a large cohort of patients and a pseudo‐stimulation group deserves further investigation.

## Author Contributions

T.L. and M.W. contributed to the design of the study. Y.H., J.G., and J.D. contributed to preparing the figures and the writing. Y.H., Y.Z., J.G., X.W., Q.D., and Y.Y. contributed to analyzing the data. J.W. contributed to reviewing the EEG data. L.C. contributed to reviewing the neuropsychological assessment data. H.Y., M.W., H.W., and J.R. provided valuable comments on the article.

## Ethics Statement

Approval for this study was obtained from the Ethics Committee of Sanbo Brain Hospital, Capital Medical University.

## Consent

Written consent for publication was acquired from all authors.

## Conflicts of Interest

The authors declare no conflicts of interest.

## Data Availability

The data that support the findings of this study are available from the corresponding author upon reasonable request.
